# Association Between Patient Demographic Characteristics and Devices Used to Access Telehealth Visits in a US Primary Care Network

**DOI:** 10.1001/jamahealthforum.2022.2932

**Published:** 2022-09-02

**Authors:** Allison Hare, Srinath Adusumalli, Ateev Mehrotra, Eric Bressman

**Affiliations:** 1 Department of Medicine, Perelman School of Medicine, University of Pennsylvania, Philadelphia; 2 Leonard Davis Institute of Health Economics, University of Pennsylvania, Philadelphia; 3 Department of Health Care Policy, Harvard Medical School, Boston, Massachusetts; 4 Division of General Medicine, Beth Israel Deaconess Medical Center, Boston, Massachusetts; 5 Department of Medicine, Corporal Michael J. Crescenz VA Medical Center, Philadelphia, Pennsylvania

## Abstract

This cross-sectional study assesses the association between patient characteristics and use of different devices to access telehealth visits during the COVID-19 pandemic.

## Introduction

Given the surge in telehealth use during the COVID-19 pandemic,^1^ concerns exist about unequal access to telehealth among disadvantaged populations.^2,3^ Efforts to improve telehealth access may be facilitated by a better understanding of how patients access these visits. Although previous research has examined the use of audio-only vs audio-video visits, little data exist on how patients currently access telehealth visits. The aim of this study was to use data from 1 health system to assess the devices patients used for telehealth visits and how devices varied across demographic groups.

## Methods

In this cross-sectional study, we identified all adults (≥18 years) with at least 1 completed telehealth visit between December 9, 2020, and September 30, 2021, at 1 of 55 Penn Medicine primary care practices. Self-identified race and ethnicity were recorded in the electronic health record, and we linked patient zip codes to median household income data obtained from the American Community Survey.^4^

Telehealth visit data were obtained from BlueJeans by Verizon, Penn Medicine’s telehealth vendor platform, through which most of the health system’s telehealth visits are conducted. Visits conducted outside of BlueJeans were excluded, along with visits that had multiple attendees (typically a family member or caregiver), in which it could not be ascertained which attendee was the patient. We focused on the device used by each patient for the first telehealth visit.

To examine the association between patient demographic characteristics and the use of a telephone or tablet, we fit a multivariate logistic regression model using a generalized estimating equation approach to account for clustering at the clinic level. We reported odds ratios (ORs) for using a telephone or tablet vs a desktop or laptop for each demographic group. Analyses were conducted using Stata, version 16.1. The University of Pennsylvania Institutional Review Board deemed this study exempt and waived informed consent because only deidentified data were used. We followed the STROBE reporting guideline.

## Results

A total of 55 812 patients had at least 1 telehealth visit (Table). Overall, 23 243 (41.6%) patients used a desktop or laptop computer to access their visit, and 32 569 (58.4%) used a telephone or tablet. Lower rates of telephone or tablet use were observed among White patients vs Black patients (OR, 0.44; 95% CI, 0.36-0.52) and non-Hispanic non-Latino patients (this term was used in the electronic medical record; no additional information is available on patient ethnicities) vs Hispanic or Latino patients (OR, 0.73; 95% CI, 0.66-0.81). Additional factors associated with lower rates of telephone or tablet use included older age (OR, 0.79 [95% CI, 0.68-0.91] in oldest [≥80 years] vs youngest [18-29 years] age group) and higher zip code–linked median income (OR, 0.79 [95% CI, 0.70-0.90] in the highest quartile [>75%] vs the lowest [<25%]) (Table).

**Table.** Patient Demographic Characteristics and Device Type Used for Telehealth Visit

Characteristic	Patients, total No. (%)	Patients, No. (%)		Telephone or tablet use, OR (95% CI)
		Desktop or laptop use	Telephone or tablet use^a^	
No. of patients	55 812	23 243 (41.6)	32 569 (58.4)	NA
Sex				
Male	18 671 (33.5)	8728 (37.6)	9943 (30.5)	1 [Reference]
Female	37 141 (66.5)	14 515 (62.4)	22 626 (69.5)	1.25 (1.21-1.30)
Age, mean (SD), y	48.1 (17.0)	49.3 (17.7)	47.2 (16.4)	NA
Age group, y				
18-29	9114 (16.3)	3880 (16.7)	5234 (16.1)	1 [Reference]
30-39	10 772 (19.3)	4098 (17.6)	6674 (20.5)	1.16 (1.08-1.25)
40-49	10 028 (18.0)	3591 (15.4)	6437 (19.8)	1.22 (1.15-1.31)
50-59	10 421 (18.7)	4207 (18.1)	6214 (19.1)	1.02 (0.96-1.09)
60-69	8893 (15.9)	4133 (17.8)	4760 (14.6)	0.81 (0.75-0.87)
70-79	4834 (8.7)	2516 (10.8)	2318 (7.1)	0.66 (0.60-0.73)
≥80	1750 (3.1)	818 (3.5)	932 (2.9)	0.79 (0.68-0.91)
Race				
Black	13 466 (24.1)	3720 (16.0)	9746 (30.0)	1 [Reference]
White	36 086 (64.7)	16 851 (72.5)	19 235 (59.1)	0.44 (0.36-0.52)
Other or unknown^b^	6260 (11.2)	2672 (11.5)	3588 (11.0)	0.52 (0.43-0.61)
Ethnicity				
Hispanic or Latino	2443 (4.4)	773 (3.3)	1670 (5.1)	1 [Reference]
Non-Hispanic non-Latino^c^	52 344 (93.8)	21 983 (94.6)	30 361 (93.2)	0.73 (0.66-0.81)
Unknown	1025 (1.8)	487 (2.1)	538 (1.7)	0.62 (0.54-0.72)
Payer				
Commercial	40 461 (72.5)	17 069 (73.4)	23 392 (71.8)	1 [Reference]
Medicaid	4304 (7.7)	1132 (4.9)	3172 (9.7)	1.54 (1.40-1.71)
Medicare	10 635 (19.1)	4907 (21.1)	5728 (17.6)	1.19 (1.08-1.31)
VA, uninsured, or unknown	412 (0.7)	135 (0.6)	277 (0.9)	1.33 (1.11-1.58)
Zip code–linked income quartile				
≤25%	4142 (7.4)	1368 (5.9)	2774 (8.5)	1 [Reference]
26%-50%	12 890 (23.1)	4358 (18.7)	8532 (26.2)	1.03 (0.94-1.12)
51%-75%	30 377 (54.4)	13 401 (57.7)	16 976 (52.1)	0.86 (0.76-0.96)
>75%	8208 (14.7)	4038 (17.4)	4170 (12.8)	0.79 (0.70-0.90)
No information available^d^	195 (0.3)	78 (0.3)	117 (0.4)	NA

Abbreviations: NA, not applicable; OR, odds ratio; VA, Veterans Affairs.

^a^ A total of 1.7% (558) of the telephone or tablet group included patients who dialed into the BlueJeans platform on their telephone, whereas the rest accessed the visit through the standard video-enabled platform.

^b^ Other includes American Indian, Asian, East Indian, and Pacific Islander patients.

^c^ This term was used in the electronic medical record; no additional information is available on which ethnicities this category includes.

^d^ These patients were excluded from the regression analysis.

## Discussion

In a large primary care network, telephones and tablets were used for most telehealth visits, and higher rates of telephone or tablet use were seen among patients who were younger, Black or Hispanic or Latino, and living in lower-income zip codes.

These results may help inform efforts to increase access to telehealth. Use of desktops or laptops depends on access to wired broadband, whereas smartphones and many tablets can use either wired or wireless broadband. It was unclear whether the higher rates of telephone or tablet use observed among disadvantaged populations were associated with limited wired broadband and computer access or patient preference. Nonetheless, our findings suggest that beyond the recent major federal investment in wired broadband,^5^ other opportunities (eg, financial support for cellular data plans,^6^ expansion of 5G networks into underserved communities) may help support patients in accessing telehealth services.

Limitations of this study were the focus on a single health system, exclusion of visits conducted outside its telehealth vendor platform, and inclusion of only those patients who successfully connected to a visit. Future interventions to bridge the digital divide could benefit by paying attention to the devices patients use to access virtual care.
